# Draft genome sequence of a novel hydrogenogenic CO-oxidizing bacterium that is phylogenetically closely related to the family Thermicanaceae

**DOI:** 10.1128/mra.00672-26

**Published:** 2026-07-24

**Authors:** Jota Suzuki, Yoshinari Imaura, Ryoma Kamikawa, Takashi Yoshida

**Affiliations:** 1 Graduate School of Agriculture, Kyoto University 12918, Kyoto, Japan; 2 Graduate School of Agricultural and Life Sciences, The University of Tokyo, Tokyo, Japan; DOE Joint Genome Institute, Berkeley, California, USA

**Keywords:** hydrogenogenic, carbon monoxide, Thermicanaceae

## Abstract

An anaerobic, thermophilic hydrogenogenic carbon monoxide (CO)-oxidizing *Bacillota* bacterium, 3446A, was isolated from freshwater pond sediments in Kyoto, Japan. The 3.11-Mbp draft genome sequence (guanine-cytosine content, 50.4%) encodes CODH–ECH genes, consistent with hydrogenogenic CO oxidation. Phylogenomic analysis suggests that *Bacillota* bacterium 3446A represents a novel genus-level lineage within the family Thermicanaceae.

## ANNOUNCEMENT

**Fig 1:**
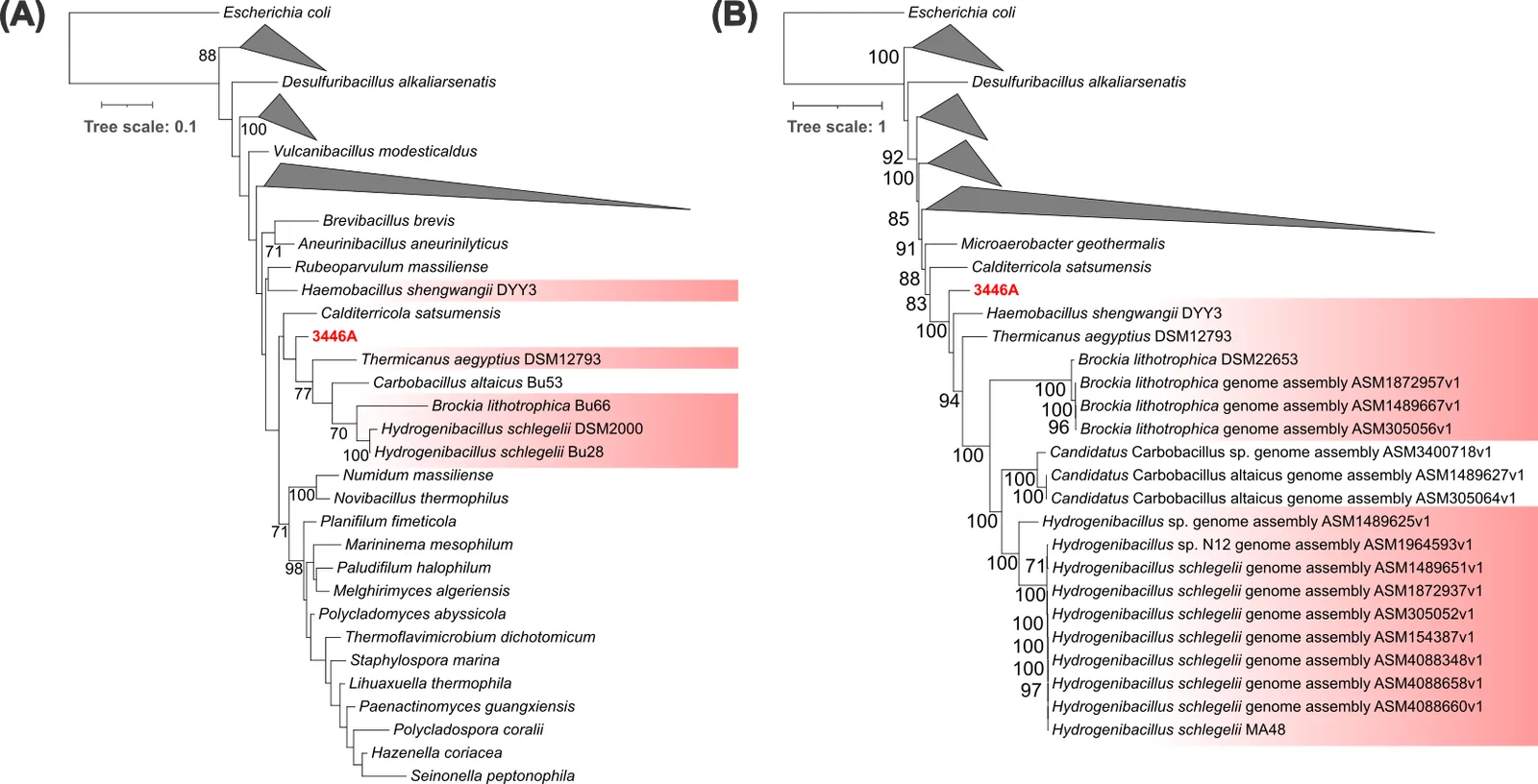
Maximum-likelihood phylogenetic trees of *Bacillota* bacterium 3446A (**A**) 16S rRNA gene analysis. The data set included all type strains of the type species within the class Bacilli. The tree was inferred using the TVMe+I+R7 model selected by ModelFinder implemented in IQ-TREE (v2.2.2.6). *Escherichia coli* was used as the outgroup. Bootstrap support values were calculated with 100 replicates, and values ≥70% are shown at nodes. Clades not directly relevant to this study were collapsed for visualization. For the Thermicanaceae-related lineages highlighted in red, all publicly available rRNA genes were included. The scale bar represents substitutions per site. (**B**) Concatenated marker gene analysis. The data set included all type strains of the type species within the class Bacilli. The tree was inferred using the LG+C10+F+G model. The statistical support for each bipartition was calculated by a 100-replicate nonparametric ML bootstrap analysis. For the bootstrap analysis, we applied the LG+C10+F+G+PMSF model (the ML tree inferred was used as the guide). Bootstrap values ≥70% are shown at nodes. Clades not directly relevant to this study were collapsed for visualization. For the Thermicanaceae-related lineages highlighted in gray, all publicly available MAGs were included. All other details are the same as those described for the 16S rRNA gene analysis.

The family Thermicanaceae in *Bacillota* comprises *Thermicanus*, *Hydrogenibacillus*, *Brockia*, and *Haemobacillus*, which form a monophyletic clade (1–5). Here, we report on the *Bacillota* bacterium, 3446A, a novel hydrogenogenic carbon monoxide (CO)-oxidizing bacterium from freshwater pond sediments in Kyoto, Japan, which is phylogenetically related to Thermicanaceae (Fig. 1).

The sediment was collected at 16 cm depth from a freshwater pond in Kyoto, Japan (35°01′50″N, 135°47′12″E) using a polyvinyl chloride pipe. Sediment (2.5 g) was inoculated into 5 mL modified B medium, prepared as previously described (6), and incubated at 65°C under 20% CO and 80% N_2_. Isolation was achieved through three successive rounds of clonal isolation by dilution-to-extinction up to 10⁸-fold under identical conditions. After 1 day of final incubation, gas chromatography (Shimadzu) confirmed CO depletion (0.912 μmol/mL) and H_2_ production (0.119 μmol/mL).

For whole-genome sequencing, the isolate was grown in 100 mL of modified B medium, and genomic DNA was extracted using the DNeasy Blood and Tissue Kit (Qiagen). A library was prepared with the MGIEasy FS DNA Library Prep Set (8-min enzymatic fragmentation) and MGIEasy DNA Adapters-96 (Plate) Kit, circularized with the MGIEasy Circularization Kit, and sequenced on DNBSEQ-G400 using the DNBSEQ-G400RS High-Throughput Sequencing Kit (MGI Tech) in paired-end mode (2 × 200 bp) at Bioengineering Lab. Co., Ltd., yielding 16,114,013 paired-end reads.

All genomic analyses used default parameters unless stated. Reads were adapter-trimmed and quality-filtered using fastp ver 0.23.2 (7, 8), resulting in 14,389,190 clean reads. These were assembled using SPAdes v3.15.4 (9), and scaffolds shorter than 500 bp were removed. The remaining scaffolds were annotated using the DFAST server v1.3.6 (10). The draft genome of *Bacillota* bacterium 3446A consists of 107 scaffolds, with an average genome coverage of 807× and an N50 length of 176,770 bp, a total length of 3,117,974 bp, a guanine-cytosine (GC) content of 50.4%, and 3,074 coding sequences (CDSs). Phylogenetic analyses based on both partial 16S rRNA gene sequences and concatenated conserved marker genes were reconstructed using IQ-TREE v2.2.2.6 (11), as described in the Fig. 1 legend, and placed *Bacillota* bacterium 3446A near Thermicanaceae, including *Thermicanus aegyptius* DSM12793, *Hydrogenibacillus schlegelii* strain MA48, *Brockia lithotrophica* strain DSM22653, *Haemobacillus shengwangii* strain DYY3, and *Candidatus* Carbobacillus altaicus (12). Average Nucleotide Identity/Average Amino Acid Identity (ANI/AAI) between 3446A and the established Thermicanaceae species calculated using JSpeciesWS (13) and FastAAI (14) were 64.7–67.3%/46.9–55.5% below species (95%) and genus (68–72%) thresholds (15–18), indicating that *Bacillota* bacterium 3446A represents a novel genus-level lineage related to Thermicanaceae.

Consistent with the cultivation results, BLASTp-based searches (19) revealed the coexistence of CODH and ECH genes in *Bacillota* bacterium 3446A; the surrounding gene regions were extracted using SeqKit v2.3.0 (20) and reannotated using RAST (21). This supports its capacity for hydrogenogenic CO oxidation (22, 23), a trait absent in its closest relatives. *Bacillota* bacterium 3446A also encodes a complete aerobic respiratory chain (complexes I–IV), glycolysis, and the TCA cycle, but lacks the nitrate- or sulfate-respiration gene, suggesting a facultative anaerobic or microaerophilic heterotrophic lifestyle.

## Data Availability

The metagenomic reads and assembled scaffolds have been deposited in the DDBJ Sequence Read Archive and DDBJ/ENA/GenBank under BioProject accession number PRJDB40626 and BioSample accession number SAMD01831029. The scaffold accession numbers are BAAKIQ010000001–BAAKIQ010000107. Raw sequence reads are available under accession number DRR1052672.

## AFTER EPUB

*[This article was published on 24 July 2026 with an error in the title. The title was corrected in the current version, posted on 29 July 2026.]*
